# Retrospective study on pattern and outcome of management of sigmoid volvulus at district hospital in Ethiopia

**DOI:** 10.1186/s12893-019-0561-1

**Published:** 2019-08-09

**Authors:** Gersam Abera Mulugeta, Seble Awlachew

**Affiliations:** Jimma University, Jimma, Ethiopia

**Keywords:** Sigmoid volvulus, Outcome, Primary resection and anastomosis

## Abstract

**Background:**

Sigmoid volvulus is the commonest cause of large bowel obstruction in many regions of the world. Its prevalence varies greatly geographically. In Ethiopia, the disease is the commonest cause of emergency admissions due to intestinal obstruction. However, few studies have been conducted discussing the management outcome in Ethiopia and Africa. This research was conducted to assess the pattern & management outcome of acute sigmoid volvulus at a district hospital in South-west Ethiopia.

**Methods:**

A facility based retrospective cross-sectional review of surgical records was done to identify patients who had acute sigmoid volvulus. Data was collected using structured questionnaire by three pre trained data collectors. The collected data was checked for its completeness, and then entered, edited, cleaned and analyzed using Statistical Package for the Social Sciences (SPSS) version 22.0. For all statistical significance tests the cut-off value set was *P* < 0.05.

**Result:**

A total of 131 patients were managed for acute sigmoid volvulus. 108 (82.4%) were men with a male to female ratio of 4.7:1. The hospital prevalence of acute sigmoid volvulus was 27.9%. Majority (42%) of the patients were in the 6th decades of life. Abdominal pain, abdominal distention & inability to pass feces & flatus were the predominant presenting compliant while abdominal distention was the dominant physical finding in all of the patients. Ninety-seven patients (74%) had viable bowel obstruction of which 29 patients had successful rectal tube deflation. The remaining 68 patients were managed operatively by either primary resection & anastomosis (62 patients) or derotation alone (6 patients). Thirty-four patients had gangrenous bowel obstruction and were managed by either primary resection and anastomosis (16 patients) or Hartman’s colostomy (18 patients). Six patients died of which 5 had primary resection and anastomosis (2 for viable and 3 for gangrenous bowel obstruction).The predominant postoperative complication was wound infection in 11(10.7%) patients. Factors associated with unfavorable outcome were female sex, primary resection & end to end anastomosis and presentation of illness more than 24 h.

**Conclusion:**

The most common management was primary resection and anastomosis. The overall mortality rate was 4.5% and the mortality rate related to primary resection and end to end anastomosis was 6.4%. Mortality rate was higher in those patients who had resection and anastomosis for gangrenous bowel compared to those who had viable bowel (19% vs 3%). Generally factors associated with poor outcome were duration of illness, primary resection and anastomosis and being female.

## Background

Sigmoid volvulus is one of the frequently occurring surgical diseases known to man since time immemorial [1]. It is an abnormal twist of the sigmoid colon on its own mesenteric axis more than 180 degrees. It initially results in obstruction of the intestinal lumen and, if not managed quickly, obstruction of the mesenteric vessels causing bowel ischemia [2]. Sometimes the small bowel gets entrapped along the sigmoid volvulus and form a knot. This is termed as Ileo-sigmoid knotting [3].

The prevalence of sigmoid volvulus as a cause of large bowel obstruction varies greatly geographically, from 1 to 7% in the United States [4] to nearly 80% in the Andes [5]. The highest incidence from Africa is reported from Ethiopia where it accounted for 56% of patients with intestinal obstruction [6]. It is more common among old men and very rare in children. The typical patients are old aged men with chronic constipation and on psychotropic medication [7].

The pathophysiology and underlying mechanism responsible for the wide geographic variation in sigmoid volvulus is poorly understood but may be related to high altitude, endemic infections, diet, or cultural factors. Other etiological factors of sigmoid volvulus include anatomic variation, chronic constipation, neurological disease, and mega colon [8, 9].

In the acute stage, pain, constipation and abdominal distension are the commonest clinical features. In gangrenous cases, blood may be seen in the rectum on digital examination. Diagnosis is made on clinical and radiological findings [10].

Sigmoid volvulus with viable bowel is commonly treated by endoscopic decompression using a long rectal tube via sigmoidoscopy. Later a delayed elective sigmoid resection is done through open or laparoscopic approaches [11]. In case of gangrenous sigmoid volvulus, the management involves adequate and prompt fluid resuscitation followed by laparotomy and decompression of proximal bowel with urgent resection of the gangrenous segment. In many centers, resection is followed by Hartmann’s procedure but primary resection with anastomosis can be considered in the absence of absolute contraindications. Contraindications include shock, local purulent infection, fecal contamination, and perforation of necrotic bowel. Sigmoiopexy and mesosigmoidoplasty are additional options of operative management for uncomplicated sigmoid volvulus. These have been shown to be reliable methods of treatment in both emergency and elective settings [7, 12–15].

As mentioned earlier, even though sigmoid volvulus is the main cause of intestinal obstruction in Ethiopia [6], local studies on its management outcome are lacking. The aim of this study is to assess the pattern of patients who had acute sigmoid volvulus and outcome of their treatment at a district hospital in Ethiopia.

## Methods

### Study design

The study design was facility based descriptive retrospective cross-sectional study. This study included patients who had treatment for acute sigmoid volvulus at Metu Karl Referral Hospital (MKRH) from September 2012 to August 2017. MKRH is a district hospital found in Mettu town, oromia region, south west Ethiopia, 600 km from Addis Ababa. MKRH is a government hospital established by Swedish missionaries in1939. It is the only referral hospital in the zone serving catchment population of 1.6 million people. It has 216 beds, out of which 65 are surgical.

### Study population, data collection and analysis

The study population were patients who were managed for large bowel obstruction due to acute sigmoid volvulus during the study period. Data of these patients was obtained from surgical ward, out-patient department (OPD) and major operation registry books. Charts of these patients were collected from card room and checked for inclusion and exclusion criteria. Patients, whose charts were lost from card room or had incomplete data (information), were excluded from the study. Twenty-one cases were excluded by this exclusion criteria. Data was collected using a data collection tool, which was adopted from other similar studies. Training was given for the data collectors regarding the purpose of the study and the procedures to be followed during data collection by the principal investigator, who was also supervising them during the data collection time from November 2016–January 2017. The collected data was coded, cleaned and entered into SPSS version22.0 for analysis. Association between each dependent and independent variables was assessed by binary logistic regression with *p*-value < 0.05 considered as significant association. To confirm statistical significance variables were then entered in to multiple regression and statically significant variables were taken at 95% CI.

### Ethical clearance

The ethical issue of this study was approved by the ethical committee of Jimma University, College of Public Health & official permission to undertake the study was obtained from the MKRH. Privacy & confidentiality of patients was maintained during data collection.

## Results

A total of 469 patients with bowel obstructions were admitted to surgical ward of MKRH in the 5 year study period. Two hundred eight of them had large bowel obstruction of which 152(73%) were due to acute sigmoid volvulus. One hundred thirty-one patients were included in this study (data retrieval rate of 86.2%). Majority of them were men (82.4%; male to female ratio of 4.7:1) and above 60 yrs. (42%). The mean age was 69 yrs. & ranged from 25 to 88 years. There were no patients in the age range of 10-19 yrs. Majority of the patients came from rural areas (71.8%).

More than half of the patients (54.2%) came to hospital 24 h after their onset of illness (duration of illness ranged from 6 h to 5 days). Women (72.3%) and those from rural areas (63.8%) tended to present to hospital later than 24 h in comparison to men (50.9%) and those that were from urban (29.7%) areas.

All patients had colicky abdominal pain, abdominal distention, and inability to pass feces & flatus as their main presenting complaint. Vomiting & constipation was noticed in 26(19.8%) & 44(33.5%) patients respectively. On physical examination abdominal distention was a finding in all the patients. Twenty one (16%) patients presented with vital sign derangement, 29 (22.1%) had abdominal tenderness, 91(69.4%) presented with empty rectum, while 13(9.9%) had blood on exam finger during digital rectal examination. Total WBC count was determined for 43 (32.8%) patients out of which a raised WBC count (> 10,000 cells/mm3) was noted in 17(39.5%). Eighty-two patients (62.5%) had plain abdominal x-ray performed prior to management. The other patients were not investigated because of unavailability of lab or radiology resources.

Twenty-nine (22.1%) patients were managed non-operatively by non-endoscopic rectal tube deflation due to lack of sigmoidoscope while the remaining 102 (77.9%) patients were managed operatively, due to failure of rectal tube deflation, suspicion of ischemic bowel or due to surgeon preference. Among those managed operatively 34 patients (33.3%) had gangrenous bowel.

**Fig. 1:**
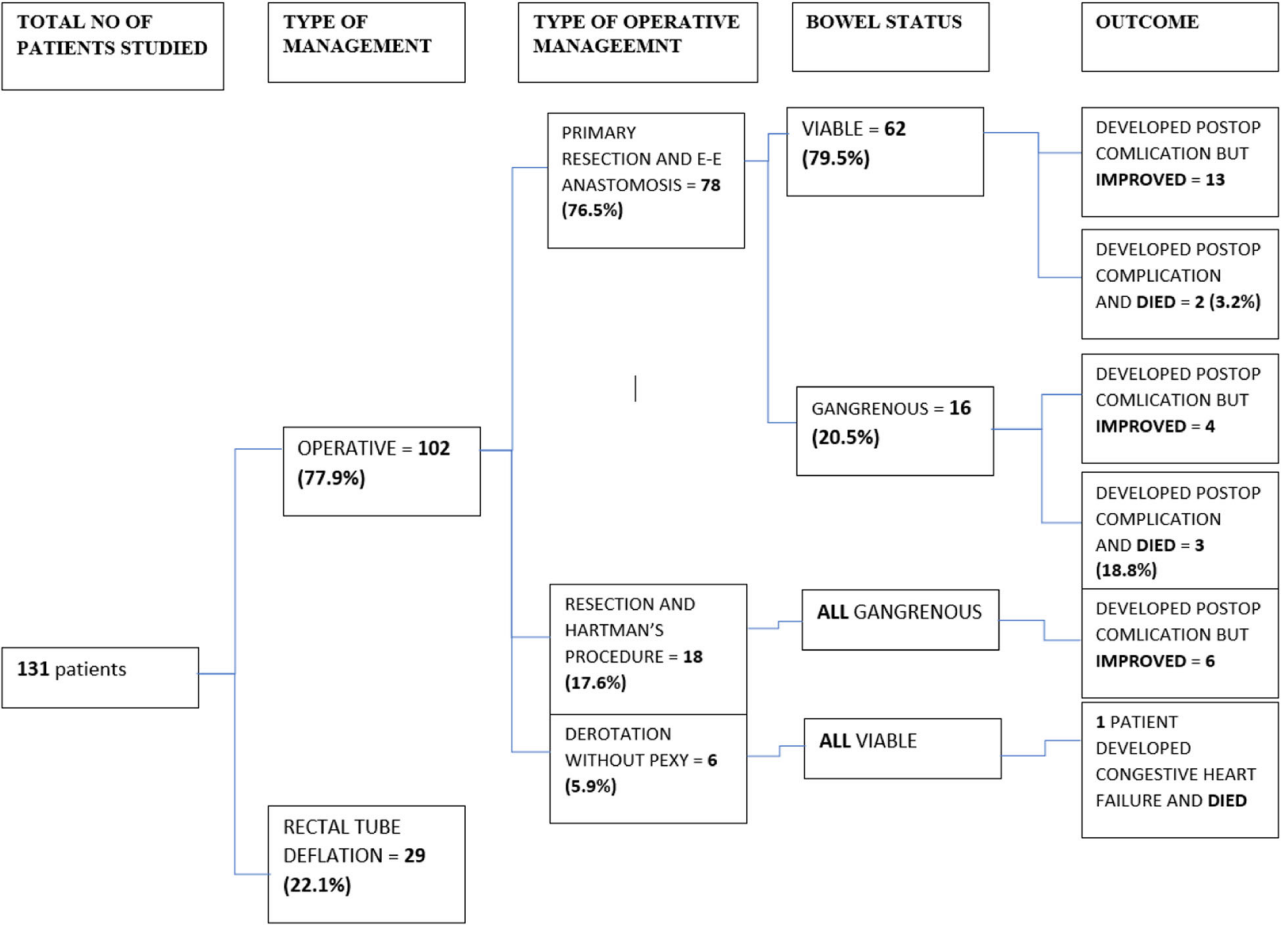
Flowchart showing the management and outcome of patients who came to MKRH with sigmoid volvulus from September 2012 to Aug 2017

From those patients managed operatively, 78 (76.5%) patients underwent primary resection & end to end anastomosis. Eighteen (18.7%) patients underwent Hartman’s procedure for gangrenous bowel obstruction. Six (5.9%) patients underwent operative derotation due to failed rectal tube deflation (Fig. 1).

Among those patients who had primary resection and anastomosis, 22 (28.2%) patients had unfavorable outcome, i.e. developed post operative complications. These complications occurred in 43.8% of those with gangrenous bowel while only 24.1% of patients with viable bowel developed postoperative complications. Five patients (6.4%) died after primary resection and anastomosis of which 3 were having gangrenous bowel (Fig. 1).

A third (1/3) of the Hartman’s procedure patients developed postoperative complications but all improved. One patient died after operative sigmoid derotation secondary to congestive heart failure due to previous cardiac problem.

About 1/5 (18.8%) of patients, who came with gangrenous bowel and were managed by primary resection and anastomosis, died during the same admission while none died after Hartman’s procedure. Only 2 (3.2%) patients died after primary resection and anastomosis for acute viable sigmoid volvulus.

All patients managed non operatively by non-endoscopic rectal tube deflation had favorable outcome. The length of hospital stay ranged from 3 to 38 days having an average length of stay of 13.5 days.

Overall 29 (22.1%) patients had unfavorable outcome. The commonest post operative complication was wound infection 13 (12.7%) followed by wound dehiscence 6 (5.9%), anastomosis leakage 4 (3.7%), and post operative intra abdominal abscess 2 (1.9%). Re-laparotomy was performed for 12 patients and the commonest was wound dehiscence. There were a total of six deaths (Mortality Rate – 4.5%).

After removing confounding factors with multivariate regression analysis, female sex (AOR = 3.97(1.19,12.2), CI 95%, *p* = .025), duration of illness more than 24 h (AOR = 3.47(1.2,10.4), CI 95%, *p* = 0.027), and primary resection and anastomosis (AOR = 3.92 (. 242,12.4,), CI 95%, *p* = .020) were identified to be significantly associated with unfavorable outcome. The other variables were not significantly associated with the outcome of patients.

## Discussion

Sigmoid volvulus is not a common disease entity in the USA but it is one of the commonest cause of large bowel obstruction in many regions of the world. Ethiopia has sigmoid volvulus as its most common cause of large bowel obstruction, which is seen in this study (73%) and previous studies (Gondar 56%) [6]. The exact etiology is not well known but literature shows that dietary habits, cultural stool withholding behavior and chronic constipation can result in a redundant sigmoid which can twist along its narrow mesenteric base and cause bowel obstruction [8].

Sigmoid volvulus occurred less commonly in females (this study 4.7:1) similar to studies done in Turkey 3.2:1, West Africa 14.3:1, Ethiopia 13.5:1, and Uganda 5.3:1which is proposed to be due to their capacious pelvic cavity and soft abdomen [6, 16–18]. Even though female patients in our study were less commonly affected, they presented later than 24 h and their outcome was poor. But it is difficult to make any conclusion because the number of female participants in the study were very few. Other studies have not showed such difference. Age-wise older patients were more commonly affected (mean age – 69 yrs), similar to other studies [19]. This indicates that redundancy of the sigmoid develops after long period of time due to continuous exposure to predisposing factors.

An obstructed bowel develops ischemia and perforation due to abnormal and prolonged distension of the twisted loop unless it is managed urgently [19, 20]. Most patients in this study (55%), similar to other study done in Ethiopia [6], came to hospital after 24 h (ranged from 6 h – 5 days) and two-third of these patients developed gangrenous bowel. This may be because patients coming from rural areas (71.8%) may have to travel long distance and may prefer traditional remedies before coming to hospital which may delay their presentation to hospital. Multivariate regression analysis showed that patients who presented after 24 h of duration of illness were three times more likely to develop unfavorable outcome compared with patients who presented with in 24 h (*p* value - < 0.05).

Overall, Ninety-seven patients (74%) had viable bowel obstruction of which 29 patients had successful rectal tube deflation. Studies done in Sudan and Ethiopia (Gondar, Atat and Tikur Anbessa) had also similar rate of viability of the sigmoid, 73, 76, 68 and 64% respectively. In the study done in Italy by Cirocchi 14 out of 23 patient (61%) had viable sigmoid volvulus who were grouped as sub occlusive patients [6, 19, 21–23].

According to studies done in Pakistan, Uganda, and Ethiopia, primary resection and anastomosis was the mode of treatment for 100, 74, and 94% of their patients respectively. In our study 78% of the patients had primary resection and anastomosis which is comparable to these studies with an overall mortality rate of 6.4%. The mortality rates in Pakistan, Uganda and South Africa after primary resection and end to end anastomosis for their patients were 0, 18 and 4.5% respectively [18, 24, 25]. According to the study done at Polokwane-Mankweng hospital, the highest mortality (17%) occurred in cases of resection and primary anastomosis of gangrenous sigmoid colon which is similar to our study (19%). Comparatively the mortality in patients with resection and anastomosis for viable bowel had lower mortality (3%) [25]. Current literatures advocate primary resection and anastomosis as safe and effective way of treatment especially for the viable sigmoid volvulus [26]. Operative management outcome for sigmoid volvulus with primary resection and anastomosis depends on the viability of the bowel. Other ways of treatment include rectal tube deflation and elective resection or sigmoidopexy [27].

The most common post operative complication in this study was wound infection (12.7%) followed by wound dehiscence(5.8%), anastomotic leakage(3.9%) and intra abdominal abscess (1.9%). A study in Pakistan revealed a similar rate of wound infection(18.2%) and pelvic abscess(9.1%) [24].

The duration of hospital stay in this study ranged from 3 to 38 days(mean 13.5) similar to study done in Pakistan (9-24 days with mean stays of 12 days) [24].

## Conclusion

The prevalence of sigmoid volvulus was 73% in this study. Majority of patients were above 6th decades of life and male. Most patients came from rural areas and duration of presentation was more than 24 h. Generally factors associated with poor outcome were duration of illness, primary resection and anastomosis and being female. The most common management was primary resection and anastomosis. The overall mortality rate was 4.5% and the mortality rate related to primary resection and end to end anastomosis was 6.4%. Mortality rate was higher in those patients who had resection and anastomosis for gangrenous bowel compared to those who had viable bowel (19% vs 3%). Primary resection and anastomosis seems an acceptable way of treatment for viable sigmoid volvulus in resource limited countries like ours.

## Data Availability

The datasets used and/or analyzed during the current study are available from the corresponding author on reasonable request.

## Abbreviations

IESO: Integrated Emergency Surgical Officer
MKRH: Metu Karl Referral Hospital
OPD: Out Patient Department
SPSS: Statistical Package for the Social Sciences
WBC: White Blood Count MSC Master of Science
